# Potential interactions between traditional Chinese medicine and osimertinib: a Case Report

**DOI:** 10.3389/fphar.2025.1596913

**Published:** 2025-06-30

**Authors:** Min Zhang, Huiwen Ma, Wanyi Chen, Zhongzhen Yuan

**Affiliations:** ^1^ Department of Pharmacy, Chongqing University Cancer Hospital, Chongqing, China; ^2^ Department of Medical Oncology, Chongqing University Cancer Hospital, Chongqing, China

**Keywords:** osimertinib, traditional Chinese medicine (TCM), drug-drug interaction, non-small cell lung cancer, therapeutic drug monitoring

## Abstract

Osimertinib, a third-generation EGFR tyrosine kinase inhibitor, played a crucial role in the treatment of EGFR-mutated non-small cell lung cancer. This case firstly reported a case of drug-drug interaction between traditional Chinese medicine (TCM) and osimertinib. A 57-year-old female was diagnosed with stage IV lung adenocarcinoma, who switched from gefitinib to osimertinib after disease progression. However, the carcinoembryonic antigen (CEA) results suggested biochemical recurrence after 48 months. The first osimertinib trough concentration (C_trough_) was 82.1 ng/mL, significantly lower than the FDA-reported 166 ng/mL, with concurrent elevation of the tumor marker carcinoembryonic antigen (CEA). After excluding other possible factors, the decreased osimertinib concentration might be due to the TCM taken by the patient. After adjusting the TCM prescription, the patient’s osimertinib C_trough_ was stable within the range of 145 and 228 ng/mL without significant adverse reactions and carcinoembryonic antigen levels stabilized. This case underscored the importance of monitoring drug concentrations in patients concurrently using TCM and osimertinib to optimize treatment outcomes and minimize potential drug-drug interactions.

## Introduction

Osimertinib, as a third-generation EGFR-TKI, played an important role in the treatment of EGFR-mutated non-small cell lung cancer (NSCLC) (Yuan et al., 2022). The United States Food and Drug Administration (FDA) reported osimertinib median trough concentration (C_trough_) was 166 μg/L (Verheijen et al., 2017). However, its exposure was susceptible to the influence of patients’ physiological and pathological conditions and drug-drug interactions (DDIs), resulting in significant inter-individual variability (Boosman et al., 2022). The coefficient of variation for intra-patient pharmacokinetics is 20.8%, while the inter-patient coefficient of variation is 37.5% (Boosman et al., 2022). Since 2016, the National Health Commission of the People’s Republic of China (NHC) encouraged the use of traditional Chinese medicine (TCM) as an important measure to optimize cancer diagnosis and treatment models (Author Anonymous, 2016). TCM had been widely used to enhance immunity, alleviate adverse reactions, and assist anti-tumor treatment in cancer patients (Chen et al., 2015; Li et al., 2022; Yu et al., 2022). It was found that most TCM extracts inhibited CYP450 mediated-metabolism of at least three isozymes (ranging from 25%–100%) (Foster et al., 2002). Osimertinib was primarily metabolized by hepatic CYP3A4/5 enzymes and transported by efflux transporters such as P-glycoprotein (P-gp) and breast cancer resistance protein (BCRP) (Yokota et al., 2022). However, due to the complex composition of TCM prescriptions, potential pharmacokinetic and pharmacodynamic interactions existed with concurrently used Western drugs (Li et al., 2022). This article is the first to report a case of drug interaction between TCM and osimertinib, exploring the impact of TCM on osimertinib plasma concentration and treatment effect.

## Case description

**FIGURE 1:**
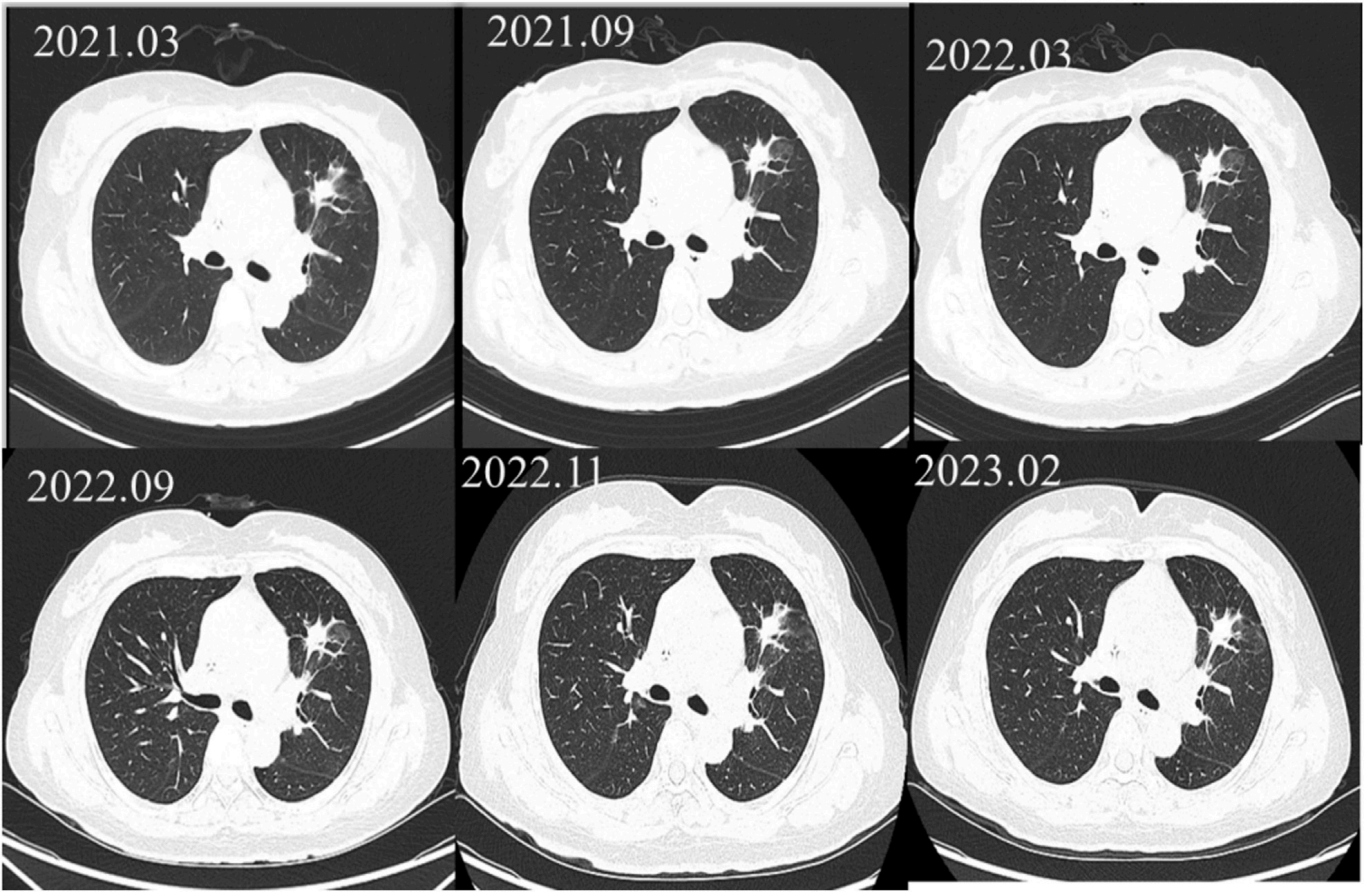
The patient’s chest computed tomography (CT) scans.

**FIGURE 2:**
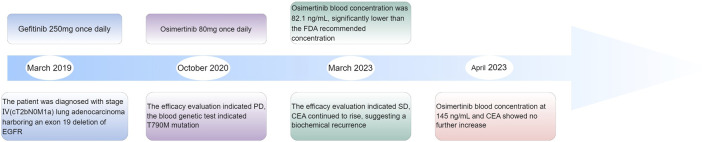
The patient’s medication adjustment timeline.

A 57-year-old female (height: 150 cm, weight: 52 kg) was diagnosed with stage IV (cT2bN0M1a) lung adenocarcinoma harboring an exon 19 deletion of EGFR in March, 2019. After 1 year and 5 months of treatment with gefitinib, the efficacy evaluation indicated progressive disease (PD), as the computed tomography of the chest suggested no significant changes in the pulmonary lesions (showed in Figure 1). The patient’s medication adjustment chart is shown in Figure 2.

The magnetic resonance imaging (MRI) of the head indicated enhancement in the anterior falx of the brain, with metastasis to be ruled out. Due to the presence of the T790M mutation in the blood genetic test, the treatment was adjusted to osimertinib 80 mg once daily in October 2020. During the period from March 2021 to February 2023, the patient exhibited stable disease (SD) (see Figure 1). However, during the regular monitoring of tumor markers, the carcinoembryonic antigen (CEA) showed an abnormal result on 11 February 2023 (19.49 ng/mL), and further increased to 28.24 ng/mL on 21 March 2023, suggesting a biochemical recurrence. The patient refused chemotherapy and, to delay the onset of osimertinib resistance, the patient was recommended increasing the dosage of osimertinib. Increasing the dosage of osimertinib might increase adverse reactions and the economic burden on patients. Osimertinib blood concentration monitoring was recommended, which could also help determine whether elevated CEA levels was related to insufficient drug concentration. Therefore, the patient was recommended taking osimertinib at a fixed time every day, and have their blood drawn half an hour before the next dose to measure the osimertinib C_trough_.

**FIGURE 3:**
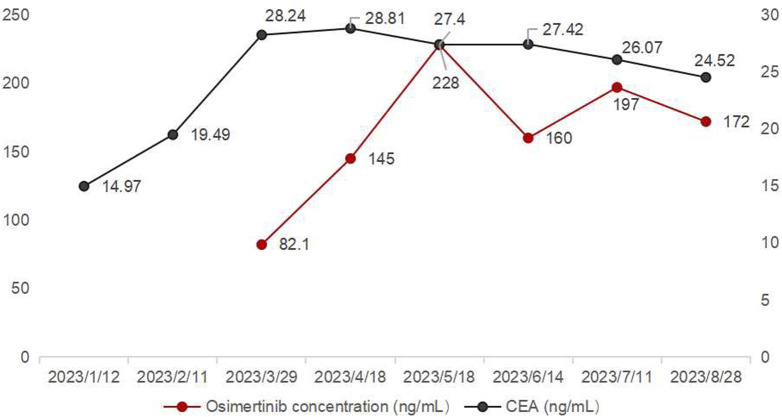
Trends of osimertinib blood concentration and carcinoembryonic antigen levels.

On 29 March 2023, the first osimertinib blood concentration was 82.1 ng/mL, significantly lower than the FDA-reported median C_trough_ of 166 ng/mL (Verheijen et al., 2017). The timing of blood sampling and potential testing errors were thoroughly investigated and subsequently excluded as causes. The patient demonstrated high adherence to the protocol (The result was shown in Supplementary Table 1). Liver and renal function tests yielded normal results. The patient did not experience nausea, vomiting, or diarrhea, and gastrointestinal abnormalities were ruled out based on routine stool examination (The result was shown in Supplementary Table 2). Therefore, the decrease in concentration might be related to the TCM prescription used by the patient to treat cough (see Table 1). At the same time, we used the Drug Interaction Probability Scale (DIPS) to evaluate a potential interaction between TCM and Osimertinib (Horn et al., 2007). The result of this case was a score of 5, indicating that the interaction between TCM and osimertinib is probable (The result was shown in Supplementary Table 3). In the TCM prescription, Chishao (*Paeonia lactiflora*), Ezhu (*Curcuma zedoaria*), Fuchaobaizhu (*Atractylodes macrocephala*), Danggui (*Angelica sinensis*), and Gancaopian (*Glycyrrhiza uralensis*) might act on P-gp and CYP3A enzymes, thereby affecting the plasma concentration of osimertinib. After communication between the clinical pharmacist and the doctor, the TCM prescription was adjusted and optimized based on the principle of syndrome differentiation and treatment (see Table 2). On 18 April 2023, a follow-up check showed osimertinib blood concentration at 145 ng/mL and CEA at 28.81 ng/mL. CEA showed no further increase, and efficacy was evaluated as SD. In multiple follow-up checks before August 2023, osimertinib blood concentration and CEA remained generally stable, and no significant adverse reactions occurred (see Figure 3).

**TABLE 1:** The patient had recently taken traditional Chinese medicine prescription.

NO.	Pinyin Name (Latin name)	Dose(g)	NO.	Pinyin Name (Latin name)	Dose(g)
1	Danggui (*Angelica sinensis*)^*#^ (Wu et al., 2016; Li et al., 2010)	15	14	Sanleng (*Sparganium stoloniferum*)	10
2	Gancaopian (*Glycyrrhiza uralensis*)^*^ (Li et al., 2010; Wang et al., 2011)	10	15	Ezhu (*Curcuma zedoaria*)^*^ (He et al., 2010)	10
3	Chishao (*Paeonia lactiflora*)^#^ (Wu et al., 2016)	10	16	Jinqiaomai (*Fagopyrum dibotrys*)	30
4	Dihuang (*Rehmannia glutinosa*)^^^ (Wu et al., 2016)	30	17	Chaomaiya (*Hordeum vulgare*)	15
5	Fuchaobaizhu (*Atractylodes macrocephala*)	10	18	Longkui (*Solanum nigrum*)	15
6	Tianma (*Gastrodia elata*)	10	19	Dilong (*Pheretima aspergillum*)	10
7	Baihuashenshecao (*Hedyotis diffusa*)	30	20	Hongjingtian (*Rhodiola rosea*)	6
8	Danshen (*Salvia miltiorrhiza*)^#^^ (He et al., 2010; Wu et al., 2016)	30	21	Wugong (*Scolopendra subspinipes*)	1
9	Tubiechong (*Eupolyphaga sinensis*)	10	22	Quanxie (*Buthus martensii*)	3
10	Chaojiangcan (*Bombyx batryticatus*)	10	23	Diyu (*Sanguisorba officinalis*)	30
11	Donglingcao (*Rabdosia rubescens*)	30	24	Tangshuizhi (*Hirudo nipponica*)	2
12	Zhebeimu (*Fritillaria thunbergii*)^^^ (Wang et al., 2011)	10	25	Lingzhibaozi (*Ganoderma spore*)	3
13	Fabanxia (*Pinellia ternata*)^^^ (Wang et al., 2011)	15			

*indicates induction of CYP3A4 enzyme; ^ Indicates inhibition of CYP3A enzyme.

^#^Indicates P-gp induction.

**TABLE 2:** After optimizing the prescription of Chinese medicine.

NO.	Pinyin Name (Latin name)	Dose(g)	NO.	Pinyin Name (Latin name)	Dose(g)
1	Ganjiang (*Zingiber officinale*)	10	11	Chaomaiya (*Hordeum vulgare*)	15
2	Zhigancao (*Glycyrrhiza uralensis*)	10	12	Chaojineijin (*Gallus gallus domesticus*)	15
3	Renshenpian (*Panax ginseng*) ^^#ᵿ^ (He et al., 2010)	10	13	Zisugeng (*Perilla frutescens*)	10
4	Maidong (*Ophiopogon japonicus*)	30	14	Tangshuizhi (*Hirudo nipponica*)	2
5	Cuwuweizi (*Schisandra chinensis*) ^^♦^ (Wu et al., 2016; Singh and Zhao, 2017)	10	15	Danggui (*Angelica sinensis*) * (Li et al., 2010)	10
6	Hongjingtian (*Rhodiola rosea*)	12	16	Tianma (*Gastrodia elata*)	10
7	Lingzhi (*Ganoderma lucidum*)	15	17	Chaojiangcang (*Bombyx batryticatus*)	10
8	Danshen (*Salvia miltiorrhiza*)^#^^ (He et al., 2010; Wu et al., 2016)	30	18	Dilong (*Pheretima aspergillum*)	10
9	Fuchaobaizhu (*Atractylodes Atractylodes macrocephala*)^*^	15	19	Baifupian (*Aconitum carmichaelii*)	15
10	Ciwujia (*Eleutherococcus senticosus*) ^^♦ᵿ^ (He et al., 2010; Lam et al., 2022; Wu et al., 2016)	10	20	Lingzhibaozi (*Ganoderma spore*)	3

*indicates induction of CYP3A4 enzyme.

^Indicates inhibition of CYP3A enzyme.

^#^Indicates P-gp induction.

^♦^ Indicates P-gp inhibition.

^ᵿ^Indicates BCRP, inhibition.

## Discussion

During the patient’s use of the first TCM prescription, the steady-state C_trough_ of osimertinib was 82.1 ng/mL, which might reduce clinical efficacy and subsequently lead to an increase in CEA levels, consistent with previous reports (Verheijen et al., 2017; Yamazaki et al., 2023; Fukuhara et al., 2023). Previous studies have demonstrated that the exposure of multiple tumor-targeting drugs were related to their efficacy and toxicity (Verheijen et al., 2017). A Japanese clinical study found that the median progression-free survival (PFS) was significantly longer in the osimertinib C_trough_ ≥211 ng/mL group compared to the C_trough_ <211 ng/mL group (Yamazaki et al., 2023). Additionally, a prospective study by Fukuhara et al. (Fukuhara et al., 2023) reported that a median C_trough_ of 227 ng/mL for osimertinib, with patients exhibiting C_trough_ levels between 164 and 338 ng/mL experiencing longer PFS. Furthermore, osimertinib C_trough_ levels exceeding 235 ng/mL were significantly associated with more severe adverse events, which led to treatment discontinuation, dose reduction, and consequently impacted treatment efficacy (Agema et al., 2022).

The C_trough_ of Osimertinib significantly increased after modifying the components of the TCM prescription, potentially due to certain herbs in the TCM prescription affecting hepatic enzyme activity. Osimertinib was primarily metabolized by hepatic CYP3A4/5 enzymes and was transported by efflux transporters such as P-gp and BCRP (Yokota et al., 2022). When osimertinib was co-administered with a strong CYP3A4 inhibitor, its area under the curve (AUC) and Cmax increased by 24% and 20%, respectively, compared to administration alone. Conversely, co-administered with a strong CYP3A4 inducer results in decreases of 82% and 78% in AUC and Cmax, respectively (Yokota et al., 2022; Vishwanathan et al., 2018). Numerous studies had demonstrated that TCM could exert varying degrees of inhibitory or inductive effects on the activity of hepatic CYP3A enzymes and P-gp transport (Pao et al., 2012; He et al., 2010; Ioannides, 2002), which might lead to alterations in the systemic exposure of osimertinib (Xu and Li, 2019). St. John’s wort has been shown to induce the expression of CYP3A4 and P-glycoprotein, while milk thistle (Naiji) and grapefruit juice (Putaoyou) could inhibit the activity of CYP3A4 (Ioannides, 2002). Other herbal components that might influence CYP3A activity include ginseng (Renshen), garlic (Dasuan), Danshen (Danshen), and liquorice (Gancao) (Ioannides, 2002). In the first TCM prescription administered to the patient in this case, Paeonia lactiflora (Chishao) might enhance P-gp transport activity, whereas Curcuma phaeocaulis (Ezhu), stir-fried Atractylodes macrocephala (Fuchao Baizhu), Angelica sinensis (Danggui), and Glycyrrhiza uralensis (Gancao) might induce CYP3A enzyme activity (He et al., 2010; Lam et al., 2022; Lv, 2006), potentially leading to a reduction in the plasma concentration of osimertinib. The second TCM prescription eliminated Chishao (*P. lactiflora*), Ezhu (*C. zedoaria*), Gancaopian (*G. uralensis*), while adding Cuwuweizi (*Schisandra chinensis*), Ganjiang (*Zingiber officinale*), Renshenpian (*Panax ginseng*) and Ciwujia (*Eleutherococcus senticosus*), *etc.* Notably, Cuwuweizi (*S. chinensis*) has been shown to inhibit CYP3A enzymes and P-gp transport activity, significantly increasing the exposure of dual P-gp and CYP3A4 substrates, such as tacrolimus (Wu et al., 2016). After using the second TCM prescription, the patient’s osimertinib C_trough_ increased compared to the previous levels, and subsequent monitoring revealed that the osimertinib C_trough_ remained between 145 and 228 ng/mL. The trends in osimertinib blood concentration were shown in Figure 2, indicating an interaction between the TCM and osimertinib. The patient expressed satisfaction with the current treatment. This case reported the potential interaction between TCM and osimertinib. However, large-scale clinical studies and mechanism researches were needed to further confirm this interaction.

## Conclusion

TCM was widely utilized among lung cancer patients in China. However, the interaction between TCM and osimertinib remained unclear. In this case, the patient’s elevated CEA levels were associated with decreased osimertinib concentrations. Following an adjustment to the TCM prescription, osimertinib concentration increased, and the elevated CEA levels were effectively controlled. Our team recommended that osimertinib concentrations be closely monitored in patients concurrently using TCM with osimertinib.

## Data Availability

The raw data supporting the conclusions of this article will be made available by the authors, without undue reservation.
