# Supplementary material for: Potential interactions between traditional Chinese medicine and osimertinib: a Case Report
Source: Front Pharmacol. 2025 Jun 30;16:1596913. doi: 10.3389/fphar.2025.1596913 (PMC12256484; doi:10.3389/fphar.2025.1596913)
Supplement: Supplementary file 1 [file Supplementaryfile1.docx]

**Table 1. The 8-item Morisky Medication Adherence Scale**

| Questions | Yes | No | Score |
| --- | --- | --- | --- |
| 1. Do you sometimes forget to take your medicine? | 0 | 1 | 1 |
| 1. People sometimes miss taking their medications for reasons other than forgetting. Thinking over the past 2 weeks, were there any days when you did not take your medicine? | 0 | 1 | 1 |
| 1. Have you ever cut back or stopped taking your medication without telling your doctor, because you felt worse when you took it? | 0 | 1 | 1 |
| 1. When you travel or leave home, do you sometimes forget to bring along your medication? | 0 | 1 | 1 |
| 1. Did you take your medicine yesterday? | 1 | 0 | 1 |
| 1. When you feel like your illness is under control, do you sometimes stop taking your medicine? | 0 | 1 | 1 |
| 1. Taking medication everyday is a real inconvenience for some people. Do you ever fell hassled about sticking to your treatment plan? | 0 | 1 | 1 |
| 1. How often do you have difficulty remembering to take all your medications? | Never：1  Once in a while：0.75  Sometimes：0.5  Usually：0.25  All of the time：0 | | 1 |
| Response choices were yes/no for items 1–7 and a 5-point Likert response for the last item.  Scores obtained from the MMAS-8 ranged from 0 to 8, where higher scores indicated higher adherence .  Scores of 8, 6 to less than 8 and less than 6 were classified as high, medium and low adherence, respectively. | | | |
| Scale assessment time: March 29, 2023 and April 18, 2023  Total Score: 8 and 8 (High) | | | |

**Table 2. Patient's Liver, Kidney Function and Stool Routine**

| **Item** | **Results** | | **Reference range** | **Unit** |
| --- | --- | --- | --- | --- |
|  | **March 29, 2023** | **April 18, 2023** |  |  |
| **Liver function** |  |  |  |  |
| Alanine aminotransferase (ALT) | 14.50 | 17.00 | 7.00-40.00 | U/L |
| Aspartate aminotransferase (AST) | 27.70 | 27.00 | 13.00-35.00 | U/L |
| Alkaline phosphatase (ALP) | 117.20 | 90.00 | 35.00-135.00 | U/L |
| Total bilirubin | 7.28 | 8.42 | 2.00-20.00 | μmol/L |
| Direct bilirubin | 1.83 | 1.97 | 0.00-6.84 | μmol/L |
| Indirect bilirubin | 5.45 | 6.45 | 0.00-19.00 | μmol/L |
| Total protein | 83.99 | 64.9 | 65.00-85.00 | g/L |
| **Kidney function** |  |  |  |  |
| Creatinine Clearance | 89.00 | 88.00 | 88-128 | ml/min |
| **Stool routine** |  |  |  |  |
| **Color** | yellow | yellow | - | - |
| **Consistency** | **soft stool** | **soft stool** | - | - |
| **Occult Blood** | **negative** | **negative** | negative | - |
| **White Blood Cells** | **0** | **0** | 0 | /HP |

**Table 3. Drug Interaction Probability Scale**

Scale assessment time: March 29, 2023.

| Questions | Yes | No | Unk or NA | Score |
| --- | --- | --- | --- | --- |
| 1. Are there previous credible reports of this interaction in humans? | +1 | -1 | 0 | 0 |
| 1. Is the observed interaction consistent with the known interactive properties of precipitant drug? | +1 | -1 | 0 | +1 |
| 1. Is the observed interaction consistent with the known interactive properties of object drug? | +1 | -1 | 0 | +1 |
| 1. Is the event consistent with the known or reasonable time course of the interaction (onset and/or offset)? | +1 | -1 | 0 | +1 |
| 1. Did the interaction remit upon dechallenge of the precipitant drug with no change in the object drug?(if no dechallenge, use Unknown or NA and skip Question 6) | +1 | -2 | 0 | 0 |
| 1. Did the interaction reappear when the precipitant drug was readministered in the presence of continued use of object drug? | +2 | -1 | 0 | 0 |
| 1. Are there reasonable alternative causes for the event?^a^ | -1 | +1 | 0 | +1 |
| 1. Was the object drug detected in the blood or other fluids in concentrations consistent with the proposed interaction? | +1 | 0 | 0 | 0 |
| 1. Was the drug interaction confirmed by any objective evidence consistent with the effects on the object drug (other than drug concentrations from question 8)? | +1 | 0 | 0 | +1 |
| 1. Was the interaction greater when the precipitant drug dose was increased or less when the precipitant drug dose was decreased? | +1 | -1 | 0 | 0 |
| ^a^ Consider clinical conditions, other interacting drugs, lack of adherence, risk factors (eg, age, inappropriate doses of object drug).  A NO answer presumes that enough information was presented so that one would expect any alternative causes to be mentioned.  When in doubt, use Unknown or NA designationa. | | | | |
| Total Score: 5 (Highly Probable: Score >8; Probable: Score 5–8; Possible:Score 2–4; Doubtful: Score <2) | | | | |
